# When interoception helps to overcome negative feelings caused by social exclusion

**DOI:** 10.3389/fpsyg.2015.00786

**Published:** 2015-06-15

**Authors:** Olga Pollatos, Ellen Matthias, Johannes Keller

**Affiliations:** ^1^Health Psychology, Institute of Psychology and Education, University of Ulm, Ulm, Germany; ^2^Social Psychology, Institute of Psychology and Education, University of Ulm, Ulm, Germany

**Keywords:** interoception, interoceptive sensitivity, ostracism, emotion regulation, embodied cognition

## Abstract

Social exclusion affects mental and physical health. The ability to regulate emotional responses to social exclusion is therefore essential for our well-being. As individual differences in detecting bodily signals (interoceptive sensitivity, IS) have been associated with the ability of emotion regulation, we aimed at exploring whether IS fosters coping with social exclusion and flexibility in emotion regulation. The first study investigated subjective feelings and behavioral affiliation tendencies in response to ostracism using a cyberball paradigm. Sixty-nine participants were assessed who differed with respect to IS. The second study examined habitual emotion regulation processes focusing on suppression and reappraisal as well as IS in 116 participants. Main results were that the effect of ostracism on distress and behavioral affiliation tendencies were qualified by IS—being ostracized had less impact on participants with stronger IS. Furthermore, Study 2 revealed that IS was associated with habitually stronger emotion regulation strategies. We conclude that having access to bodily signals helps (IS) reducing aversive states provoked by social exclusion, probably due to the fact that IS is associated with emotion regulation strategies.

## Introduction

### Social Exclusion and Health

Social exclusion is associated with adverse effects for mental and physical health (see e.g., Zöller et al., 2010). Social exclusion, i.e., ostracism, is a ubiquitous phenomenon across the lifespan that threatens the fundamental human need to belong to a group. According to Baumeister and Leary (1995) the need to belong is a powerful, fundamental, and extremely pervasive motivation. Being ostracized elicits social pain, loneliness, anxiety and sadness (e.g., Hawkley et al., 2011). Experimental approaches to studying ostracism use behavioral manipulations that induce being excluded or ignored, and one of the most frequent methods employed is cyberball (Williams, 2006). Cyberball is a virtual ball toss game that participants play using a laptop or computer. Being socially rejected in cyberball is associated with reduction in belongingness, self-esteem, and control as well as negative affect such as anger and sadness (Sebastian et al., 2010).

While the cyberball paradigm was used in numerous studies, data on personality factors that moderate feelings of exclusion caused by cyberball are sparse: Onoda et al. (2010) recently demonstrated that participants with low self-esteem experienced increased social pain as compared to individuals with higher trait self-esteem. Other moderator variables were not reported, especially no effect of introversion-extraversion, individualism-collectivism, need for belonging, and loneliness (Williams, 2006). A possible moderator candidate stems from research on emotion processing and emotion regulation, as being socially excluded causes a variety of unpleasant feelings the ostracized person has to deal with. A salient cue signaling how comfortable people feel in social interactions is the space between them, the “interpersonal distance” (see e.g., Perry et al., 2013, 2015). As cyberball causes social pain and negative affect, the measure of interpersonal distance can be used to monitor its effect on the level later social interactions.

### Emotion Regulation and Interoception

An important human ability is to regulate such negative emotions caused by being socially excluded. There exist different strategies to regulate one’s emotions (Gross and John, 2003; Ochsner et al., 2004; Goldin et al., 2008; Pollatos and Gramann, 2012), and individuals differ in their use of emotion regulation strategies with implications for their well-being and social functioning (Gross and John, 2003; Goldin et al., 2008). Füstös et al. (2013) state that awareness of one’s emotional state is an essential variable for emotion regulation, following that this process might also be linked to the awareness of one’s bodily state. The perception of bodily changes (interoception) is a central concept in several theories of emotions (James, 1884; Damasio, 1994; Craig, 2004) that postulate a relationship between interoceptive processes and emotions.

Having these results in mind one could assume that a higher emotional arousal in emotion induction would also hamper the ability to regulate emotions. But a recent study by Füstös et al. (2013) demonstrated that the ability to perceive bodily signals (interoceptive sensitivity, IS) facilitated the downregulation of affect-related arousal when participants were instructed to use reappraisal, a common emotion regulation strategy (Gross and John, 2003; Goldin et al., 2008). Also Weiss et al. (2014) reported a positive relationship between emotion regulation abilities as assessed by questionnaire and IS.

### Social Exclusion and Interoception

Füstös et al. (2013) assume that a greater sensitivity to one’s bodily state facilitates the regulation of emotional responses. They suggested that the detection of ongoing bodily changes is easier or more accurate, and this might in turn facilitate the discrimination and regulation of different emotional states (Füstös et al., 2013). Whether these mechanisms might also facilitate the regulation of unpleasant affect elicited as response to social rejection is unclear till now. A study by Werner et al. (2013) supports this assumption: Participants took part in a discussion round and after a certain time they were excluded from the discussion. IS modulated positive and negative affect and perceived acceptance respectively rejection during exclusion. Whether the change in subjective feelings caused by exclusion leads to motivational engagement to overcome the situation and whether such a behavior is also modulated by interoceptive processes was not addressed in the former study. Ferri et al. (2013) hypothesized that IS might contribute to interindividual differences concerning social attitudes and interpersonal space; the social situation they used in the study involved an experimenter who performed movements at different distances from the participant’s hand. This setup involves no direct social interaction, but the role of interoceptive signals might be much more important in social relevant situation as implemented in the cyberball paradigm.

The idea that perceiving internal signals more precisely facilitates processes of comparisons of different internal states related to emotions and their regulation in social relevant situations would be supported when there is further evidence that interoception interacts with emotion regulation in everyday life. Emotion regulation strategies as assessed by questionnaire might therefore an interesting tool to evaluate regulation capacities in general. The idea that IS supports emotion regulation is in accordance with data from Feldman Barrett et al. (2001) who demonstrated that persons with highly differentiated emotion experience could better regulate their emotions in everyday situations.

### Interoceptive Sensitivity—Measurement

A common method to assess interoception is the ability to perceive one’s heartbeats accurately (Schandry, 1981; Critchley et al., 2004; Dunn et al., 2007; Pollatos et al., 2008). This ability can be measured by using validated and reliable heartbeat perception tasks (Whitehead and Drescher, 1980; Schandry, 1981), in which participants are instructed to perceive their own heartbeats without feeling for their pulse. There is convincing evidence that higher IS is associated with more intense feelings and higher activation of underlying brain structures during emotional stimulation (Wiens, 2005; Pollatos et al., 2007a; Dunn et al., 2010; Füstös et al., 2013). IS was also associated with cognitive functions like decision-making, selective attention or self-regulation during physical exercise (Pollatos et al., 2007b; Werner et al., 2009b; Dunn et al., 2010; Lenggenhager et al., 2013).

### Aim of the Studies

To further elucidate whether IS interacts with feelings of social exclusion and emotion regulation in general, we conducted a study on healthy participants. We hypothesized that IS is related to (1) better coping with social exclusion, (2) less motivation to engage in behavior serving to overcome these feelings of exclusion, and (3) better emotion regulation capacity and more flexibility in general. To experimentally vary social exclusion we employed a standardized paradigm (cyberball) known to affect well-being. Additionally, as ostracism causes a threat to fundamental needs (belonging, self-esteem, control, and meaningful existence, see e.g., Jamieson et al., 2010) and therefore individuals are motivated to fortify these needs, we used preferred interpersonal distance (see Perry et al., 2013) as one measure to examine behavioral tendencies to cope with the threat to the need for affiliation in the ostracism paradigm. The second study builds on the first by examining emotion regulation in general (using an established self-report measure).

## Materials and Methods—Study 1

Participants were screened for health status using an anamnestic questionnaire. Exclusion criteria were any history of any axis 1 disorders, in particular anxiety disorders or depression according to the Diagnostic and Statistical Manual of Mental Disorders as well as drug use (except of contraceptives). All participants gave their written informed consent. They received an amount of 10€ for their participation. Sixty-nine female participants (mean age 23.6, SD 3.7) were included in the main experiment. The experiment was conducted in accordance with the Declaration of Helsinki, the local ethics committee approved the study.

### Procedure

First, IS was assessed. ECG electrodes were placed to the right mid-clavicle and lower left rib cage. We used four heartbeat counting phases (varying in length) in accordance with the Mental Tracking Method suggested by Schandry (Schandry, 1981). Participants were asked to count their own heartbeats silently and to report the number of counted heartbeats at the end of the counting phase. IS was calculated according to the following transformation:

$$\frac{1}{4}\text{Σ}\left(1-(|\text{recorded heartbeats}-\text{counted heartbeats}|)/\text{recoded heartbeats}\right)$$

The mean score was 0.70 (SD 0.14).

Then, electrodes were detached and the cyberball paradigm started. As we also wanted to assess the effect of cyberball on interpersonal relations, we slightly varied the paradigm in the following manner: There were always two experimental supervisors present up to this stage of the experiment. One of them then left before the other experimental supervisor explained the cyberball paradigm to participants. Participants were randomly assigned to one of the three cyberball conditions: inclusion, social exclusion and social exclusion due to pretended technical failure. Technical failure condition was introduced to assess effects of being implicitly excluded. Participants were told that they take part in a mental visualization exercise in which they toss a ball over the internet with two other players (see Hawkley et al., 2011). Importantly, one of the other two players was the one experimental supervisor that the participants had met before. His/her photo was also depicted on the screen while the other player was unfamiliar to the participant. Participants were told that all persons involved in the procedure were connected via internet. We also took a photo of the participant in the beginning so that his/her photo was then used in the cyberball setting. Then one of three conditions took place: In the inclusion condition, participants receive the ball one-third of the time. In the social exclusion and technical failure conditions participants receive the ball only at the beginning and are then ignored. At the end of the failure condition a screen appears with an error message in which it is explained that due to connection problems of the internet the participant was no longer connected with the other players.

Subjective mood was assessed as one outcome variable; this was carried out immediately prior and directly after cyberball using the German version of the Profile of Mood States (Dalbert, 1991). Nineteen items are rated on a seven-point Likert scale comprising four aspects of negative (fatigue, depression, anger, sadness, 13 items) and positive mood (six items), translated into a negativity index (range 19–133) with higher scores reflecting greater negativity.

Feelings of exclusion were evaluated as described by (Bolling et al., 2011) using a 10-item questionnaire given to participants immediately after playing cyberball. The items were taken from the Needs Threat Scale that checks for distress following exclusion (Eisenberger et al., 2003; Hawkley et al., 2011) as adapted by Bolling et al. (2011). The Needs Threat Scale included statements about feelings of control, belongingness, and self-esteem on a Likert scale from 1 = “not at all” to 5 = “extremely.” Example items were: “During the game, I felt ignored.”; “I felt rejected”; “I felt like an outsider.” Consistent with previous research, we computed as mean needs with higher scores reflect greater needs threat (see also Hawkley et al., 2011).

We also assessed preferred interpersonal distance as suggested by Perry et al. (2013), operationalized as distance chosen between the participant and the one cyberball player who was also present in the laboratory prior to cyberball and whose photograph had been shown in the cyberball paradigm before. For the assessment of interpersonal distance, the participant and the experimental supervisor were placed directly facing each other with a start distance of three meters, and then the experimenter approached the participant until s/he said “stop” to signal a distance s/he evaluated as appropriate. This procedure was chosen very similar to the one suggested by Perry et al. (2013) in virtual reality to assess one’s preferred interpersonal distance. Our exclusion took also place on the screen with photos of the protagonist, while the later assessment of the preferred interpersonal distance was carried out in real space. The detailed instruction was to signal the distance the participant felt comfortable with in accordance to Perry et al. (2013). This preferred interpersonal distance was noted in meters.

## Study 2—Materials and Methods

Participants were screened with the procedure as applied in Study 1. They received an amount of 5€ for their participation. 116 participants (27 male) took part, their mean age was 25.6 (SD 3.2). The local ethics committee approved the experiment.

### Procedure

First, all participants filled in the German version of the Emotion Regulation Questionnaire (Abler and Kessler, 2009) developed by Gross and John (2003). The ERQ is one of the first validated instruments for the investigation of emotion regulation processes. The questionnaire tests two common regulation strategies: suppression (example item: I control my emotions by not expressing them) and reappraisal (example item: I control my emotions by changing the way I think about the situation I’m in). The German version of the ERQ consists of 10 items assessing habitual reappraisal and suppression on a seven point scale.

Afterward, the heartbeat detection task as described in Study 1 was conducted. The mean heartbeat perception score was 0.71 (SD 0.16).

### Data analyses

Study 1: We calculated three regression analyses using the following dependent variables:

a. feelings of exclusion as measured by the needs index,
b. negative feelings as measured by the negativity index, and
c. preferred interpersonal distance.

The variables a-c. as were z-standardized and then regressed on the following variables:

d. z-standardized interoceptive sensitivity,
e. dummy 1 (codes: 0 = inclusion; 1 = technical failure; 0 = exclusion),
f. dummy 2 (codes: 0 = inclusion; 0 = technical failure; 1 = exclusion),
g. the interaction involving standardized interoceptive sensitivity and dummy 1, and hour the interaction involving standardized interoceptive sensitivity and dummy 2.

In accordance to Aiken and West (1991) simple slopes analyses were applied with one SD above and below the mean IS score in order to examine possible differences between participants with high versus low IS.

Furthermore, Pearson’s correlation analyses were performed between the needs index and interpersonal distance.

Study 2: One regression analysis (forward selection) with reappraisal and suppression as predictors and interoceptive sensitivity as criterion was carried out.

## Results

### Study 1

Table 1 summarizes the needs index, the negativity index and preferred interpersonal distance evoked by the different cyberball conditions.

**TABLE 1:** **Needs index, negativity index (before and after cyberball) and preferred interpersonal distance contrasting the different conditions (*N* = 69 total; *N* = 24; SE, social exclusion; *N* = 23; SET, social exclusion, technical failure; *N* = 22; SI, social inclusion)**.

	**Mean(SD)**		
	**SE**	**SET**	**SI**
Needs index	3.9 (0.7)	3.8 (0.7)	1.4 (0.3)
Negativity index			
Before	57.8 (8.0)	56.8 (6.0)	57.1 (8.3)
After	77.5 (8.9)	74.6 (15.8)	59.0 (8.4)
Interpersonal distance (in m)	0.69 (0.17)	0.88 (0.25)	0.97 (0.14)

The first regression analysis with a. the *needs index* as criterion revealed that the criterion was explained by both dummies as well as both interaction terms [*F*(5,63) = 83.77, *p* < 0.001, *R* = 0.93, *R*^2^ adjusted = 0.86]. A significant effect of condition was reflected in significant effects of dummy 1 (*T* = 15.93, β = 0.86, *p* < 0.001) and dummy 2 (*T* = 17.35, β = 0.93, *p* < 0.001). Crucially, both interaction terms between IS and dummy 1 (*T* = –2.90, β = –0.20, *p* < 0.01) as well as dummy 2 (*T* = –3.79, β = –0.26, *p* < 0.001) were significant.

**FIGURE 1:**
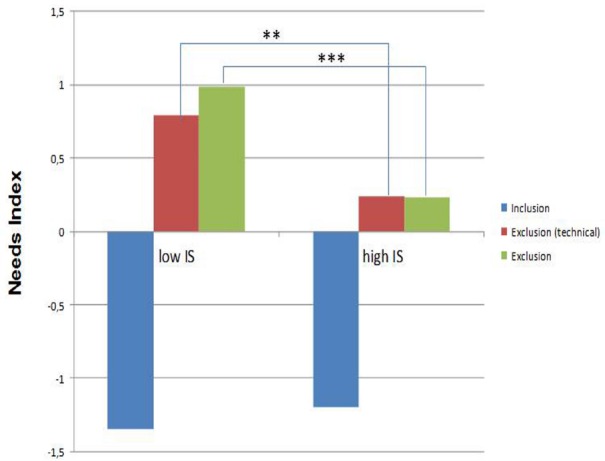
Needs index after cyberball contrasting participants with high and low IS in the three experimental conditions (*N* = 69; ***p < 0.001; **p < 0.01).

Figure 1 summarizes theses effects. Both social exclusion conditions caused greater needs index scores as compared to the mean scores in the social inclusion condition. Importantly, IS qualified the effect of ostracism. High IS (one SD above mean) was associated with less pronounced needs scores in both social exclusion conditions as compared to low IS (one SD below mean).

With respect to the b. *negativity index*, the criterion was explained by condition only [dummy 1: *T* = 4.87, β = 0.55, *p* < 0.001; dummy 2: *T* = 4.80, β = 0.65, *p* < 0.001; *F*(5,68) = 9.28, *p* < 0.001, *R* = 0.65, *R*^2^ adjusted = 0.38]. IS and both interaction terms were not significant. These effects indicate an increase in negative feelings for both social exclusion conditions (SE: mean before 57.8, mean after 77.5; SET: mean before 56.8, mean after 74.6), while no change was observed after social inclusion (mean before: 57.1, mean after 59.0).

We also obtained a significant positive correlation between the needs index and the negativity index after cyberball (*r* = 0.57, *p* < 0.001): The more participants felt excluded, the more negative feelings increased and vice versa.

**FIGURE 2:**
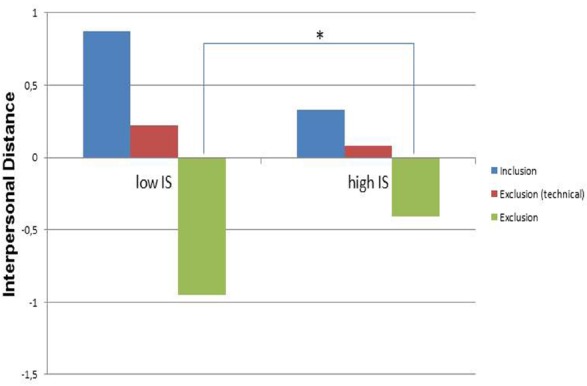
Interpersonal distance after cyberball contrasting participants with high and low IS in the three experimental conditions (*N* = 69; *p < 0.05).

The third regression analysis with *c. interpersonal distance* as criterion showed that the criterion was explained by dummy 2 and the interaction term between IS and dummy 2 [*F*(5,63) = 6.57, *p* < 0.001, *R* = 0.58, *R*^2^ adjusted = 0.29]. Interpersonal distance increased after exclusion only as reflected in the significant effect of dummy 2 (*T* = –5.31, β = –0.64, *p* < 0.001), while dummy 1 was only marginally significant (*T* = –1.85, β = –0.22, *p* = 0.07). The interaction term between IS and dummy 2 was significant (*T* = 2.09, β = 0.34, *p* < 0.05). These effects are depicted in Figure 2.

Preferred interpersonal distance was smaller after exclusion (mean distance 0.69 meters) as compared to both exclusion with technical failure explanation (mean 0.88 meters) and inclusion (mean 0.97 meters). Higher IS (one SD above mean) was associated with an decrease of interpersonal distance after social exclusion, which was more pronounced for the group with low IS (one SD below mean) and depicts the significant interaction effect.

In a last step we obtained a significant inverse correlation between the needs index and interpersonal distance of *r* = –0.40 (*p* < 0.01) indicating that a greater threat of needs was associated with a smaller interpersonal distance.

### Study 2

#### ERQ Emotion Regulation and IS

**FIGURE 3:**
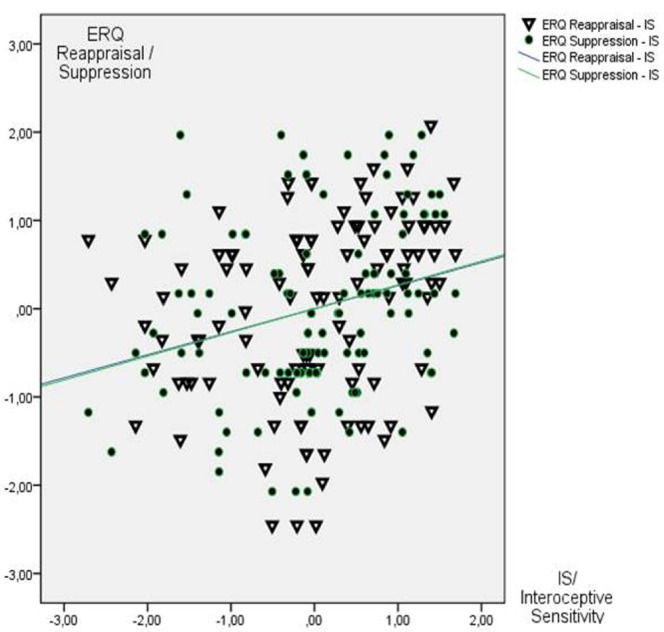
**Scatterplot between IS and both reappraisal and suppression (*N* = 116; *z*-scores depicted)**.

Mean ERQ scores were 28.2 (SD 6.2) for reappraisal and 13.2 (SD 4.5) for suppression. The consecutive regression analysis with IS as criterion and reappraisal as well as suppression as predictors revealed significant effects of reappraisal (*T* = 3.14, β = 0.27, *p* < 0.01) and suppression [*T* = 3.22, β = 0.28, *p* < 0.01; *F*(2,113) = 9.71, *p* < 0.001, *R* = 0.38, *R*^2^ = 0.15]. Higher IS was associated with both higher reappraisal and higher suppression. These effects are depicted in Figure 3 (z-standardized scores for all variables depicted).

## Discussion

Our data provide new evidence for the relevance of the perception of bodily signals regarding ostracism and emotion regulation. Being socially rejected using the cyberball paradigm is typically associated with a threat to elementary needs such as belongingness, self-esteem or control, as well as an increase in negative affect as demonstrated in former studies (Eisenberger et al., 2003; Williams et al., 2006; Sebastian et al., 2010; Hawkley et al., 2011). Importantly, IS moderated this effect: Higher IS was found to be associated lower levels of (a) distress and (b) behavioral affiliation tendencies following social exclusion. In accordance with these results IS was correlated with higher scores on the emotion regulation questionnaire.

These results are in accordance with Füstös et al. (2013) who demonstrated that IS facilitates the downregulation of affect-related arousal and corresponding neural activation when applying reappraisal as emotion regulation strategy. Our results are also in accordance to data on social exclusion in a discussion as provided by Werner et al. (2013). They suggested that individuals with high IS reduce aversive states by using somatic information for self-regulation to a greater extent. One possible explanation builds on data from Feldman Barrett and co-authors (Feldman Barrett et al., 2001): Here, emotionally differentiated participants reported a wide range of emotion regulation strategies used in daily life. We assume that IS facilitates the efficiency of different emotion regulation strategies by providing a more fine-tuned feedback of the actual emotional state. This is also the case in situations characterized by social exclusion: Both the feelings of exclusion as well as the need to affiliate as reflected by the interpersonal distance measure were less pronounced when participants were rather good in perceiving their bodily signals. We therefore suggest that IS supports the effective down-regulation of negative affect and associated bodily changes occurring during social exclusion which might lead to a lower “cost” for the self. IS assessed in one physiological system (the cardiac system) relevantly mediates emotion regulation in situations evoking physiological activity such as social exclusion. As suggested by Füstös et al. (2013), being aware of one’s bodily signals might therefore constitute a positive precondition for effective self-regulation of behavior. Supporting this interpretation, data obtained in a public speech paradigm showed that IS was associated with less self-reported state anxiety before and during such a task (Werner et al., 2009a). And also Lenggenhager et al. (2013) manipulated visceral feedback of their participants and reported that heightened feedback regarding one’s own visceral processes increased a self-centered perspective and affected drive socioeconomic exchanges accordingly. In contrast to these studies, Van ’t Wout et al. (2013) did not observe a reliably significant relationship between IS and the acceptance of unfair offers or habitual use of emotion regulation.

As expected, the need to affiliate as one coping mechanism after social exclusion was higher after social exclusion. This was only the case when no alternative explanation for the experienced social rejection was provided, as social exclusion with technical failure and social inclusion were associated with comparable distance measures. The behavioral measure—preferred interpersonal distance—was significantly smaller after social rejection and positively correlated with the experienced threat of needs. We interpret this result as an indicator for a stronger tendency to socially affiliate after rejection. Referring to the interaction between IS and interpersonal distance after social rejection, we assume that IS moderates affective processes and the coping with such negative emotions as well as the behavioral tendencies to deal with the outcome of such negative situations. In relation to our results, interoceptive processes might preserve the common resource used for self-regulatory processes. Self-regulation uses self-monitoring and affective self-reaction (Maes and Karoly, 2005) which might constitute abilities that are linked with bodily processes and the conscious feedback of these processes as operationalized by IS. Recent work supports this idea: Weiss et al. (2014) could demonstrate that IS was positively correlated with self-regulatory capacities as assessed by questionnaire. And also Koch and Pollatos (2014) showed that IS is positively correlated with greater adaptability as assessed by questionnaire in children. It can be followed that interoception helps to preserve limited resources involved in self-regulation, presumably by faster or more differentiated detection of bodily response changes occurring in significant situations such as social rejection and might help to constitute a feeling of higher control over one’s negative experiences in everyday life.

### Conflict of Interest Statement

The authors declare that the research was conducted in the absence of any commercial or financial relationships that could be construed as a potential conflict of interest.
